# Drug therapy and medication adherence in type 2 diabetes in a care facility: A cross sectional survey

**DOI:** 10.1016/j.rcsop.2022.100200

**Published:** 2022-11-04

**Authors:** Uchenna I.H. Eze, Tolulope F. Akhumi, Chinonyerem O. Iheanacho, Sule A. Saka

**Affiliations:** a Department of Clinical Pharmacy and Biopharmacy, Faculty of Pharmacy, Olabisi Onabanjo University, Sagamu, Nigeria; b Department of Clinical Pharmacy and Public Health, Faculty of Pharmacy, University of Calabar, Calabar, Nigeria

**Keywords:** Nigeria, Type 2 diabetes mellitus, Medication adherence, Glycaemic control, Health facility, Low socio-economic population, Drug therapy, DPP-4 inhibitors, Dipeptidyl peptidase (DPP)-4 inhibitors, GLP-IRAs, Glucagon-like peptide 1 receptor agonists, WHO, World Health Organisation, SPSS, Statistical package for social sciences

## Abstract

**Background:**

Adherence to medications improves glycaemic control and reduces diabetes-related morbidity and mortality.

**Objectives:**

The study assessed drug therapy for type 2 diabetes, glycaemic control and association of medication adherence with socio-demographic and clinical data, among adult diabetic patients attending a healthcare facility.

**Methods:**

Cross-sectional survey and hospital records were used to obtain data. The study included 200 adults with type 2 diabetes mellitus in a Nigerian healthcare facility. Data on patients clinical characteristics, diabetes drug therapy and medication adherence were collected, entered and anlaysed using SPSS version 24 *(P <0.05)*. Primary outcome measure was medication adherence among the patients, while secondary outcome measures was glycaemic control.

**Results:**

A total of 200 (100%) respondents participated in the study and the majority 141(70.5%) were over 60 years old. Oral medications were mostly used 187(93.5%), particularly, metformin 199(99.5%) and pioglitazone 100(50.0%), while dipeptidyl peptidase-4 inhibitors were not used at all. Patients mostly had poor glycaemic control 159 (79.5%) and majority 152(76.0%) did not practice self-blood glucose monitoring. Moderate medication adherence was predominant in the population. Class of medicine and socio-demographics were not significantly associated with medication adherence *(P>0.05*), unlike results of blood glucose self-tests (*p=0.001*).

**Conclusion:**

Oral antidiabetics, particularly metformin and pioglitazone were mostly used. Poor glycaemic control and moderate adherence were found in the patients, and medication adherence was associated with self-glucose monitoring. This emphasises the need for regular diabetes education on medication adherence.

## 1 Introduction

Diabetes is a chronic, progressive disease that is characterised by hyperglycaemia, and results in significant complications and mortality.^1^ It comprises of several sub-classification among which is type 2 diabetes mellitus. Although it is a preventable disease, high prevalence has been reported in various parts of Nigeria, with alarm of impending epidemic,^2^, ^3^, ^4^, ^5^ and this has been attributed to increased urbanization and its associated lifestyle changes.^2^

Effective management of diabetes involves pharmacological and non-pharmacological approach. Several classes of oral antidiabetic agents provide benefit for the patients, with their associated risks not overlooked. However, appropriate selection of therapeutic agent which should be targeted on both glucose and non-glucose goals, is dependent on patient's characteristics and the available clinical data.^6^ According to a joint statement by the American Diabetes Association and European Association for the study of Diabetes, the choice of medications used in the management of type 2 diabetes should be individualized, and it is dependent on a number of important factors which include; drug efficacy, risk of hypoglycaemia, side effects, effect on weight and cost.^7^

According to WHO, metformin is recommended for first line use, while the sulfonylureas are recommended for second line use, and as first line in cases where metformin is contraindicated.^1^ The insulin therapy is reserved for third line therapy. However, in economically feasible conditions, the use of medications with fewer side effects, no incidence of weight gain and hypoglycaemia and established cardiovascular safety are encouraged. Medicines in this category are dipeptidyl peptidase (DPP)-4 inhibitors, glucagon-like peptide 1 receptor agonists (GLP-IRAs) and sodium-glucose co-transporter (SGLT) 2 inhibitors.^1^^,^^7^ Therefore, the most suitable approach is individualized therapy which is based on patients factors and treatment options.^8^

Medication adherence results in improved haemoglobin AIc levels in type 2 diabetes.^9^ It is therefore, a significant measure of glycaemic control, however; high prevalence of non-adherence has been observed and associated with several factors which include; high pill burden, age,^9^ and educational level.^5^ Others are: Forgetfulness, lack of finance, relief from symptoms, dosing frequency,^10^^,^^11^ and attendance of diabetes counselling.^12^ Also, good understanding of the disease condition and management strategies by the patients dispels wrong perceptions of the disease state, enhances adherence and optimizes disease outcomes.^13^^,^^14^

Only few persons living with diabetes in Nigeria achieve glycaemic control and this is largely due to non- adherence to prescribed medicines, with very few likely to disclose their non-adherence to their healthcare provider.^15^ As a result of lower socio-economic development and under-resources, medication adherence and blood glucose control appear to be lower in Nigeria, with prevailing high burden of diabetes-complications. Among the Nigerian population, low educational and literacy level are identified factors for non-adherence to anti-diabetic medications, and these are seen in a substantial number.^5^^,^^16^ Poor medication adherence in Nigeria^16^^,^^17^ is a growing concern that undermines the benefits of clinical care in diabetes patients.

Medication adherence is a major concern in persons with type 2 diabetes, particularly in Nigeria, and this may be related to socio-demographic and clinical characteristics of the patients. Previous studies have focused on factors affecting medication adherence, while there is paucity of data on the association of clinical data and medication adherence. The aim of this study was to assess glycaemic control, medicines most commonly used and association of medication adherence with socio-demographics and clinical data in persons with type 2 diabetes mellitus in a healthcare facility, for efficient and effective management plan.

## 2 Methods

### 2.1 Study design and setting

The study was conducted using a mixed method comprising retrospective (hospital records) and cross-sectional survey. It was conducted in Ijebu-Ode general hospital, a secondary health facility in Southwest Nigeria. The facility is at the middle of the 3-tier hierarchy of hospitals in Nigeria, serving as a referral centre for primary health centres and makes referrals to tertiary health facilities when necessary. It is located in a sub-urban area of the country with limited industrialisation. Residents of this region are mostly of low and middle income.

### 2.2 Study population

The study was conducted among adults of over 30 years old who had type 2 diabetes and visited the diabetes clinic in the general hospital, Ijebu Ode – Southwest Nigeria. Persons with type 2 diabetes who were either out-patients or in-patients were included in the study. However, newly diagnosed patient (<3 months) were excluded from the study. Patients from whom informed consents were not obtained were also excluded.

### 2.3 Sample size calculation

Sample size calculation was based on estimated patient turnout in the hospital. Weekly average number of in-patients and out-patients with type 2 diabetics was 28, and a calculated study population of 336 (estimated diabetic patients in 12 weeks) was derived for the hospital. Using an online sample size calculator,^18^ a sample size of 180 was calculated and a 5% attribution was included. A sample size of 189 was obtained and this was rounded up to 200.

### 2.4 Sampling technique

Sampling was done in two phases involving a cross-sectional survey of 200 consecutively recruited in-patients and out-patients who visited the endocrinology clinic over a 12-week period, and a corresponding review of the patients' case files. Information detailing tests, evaluations, results and physicians' documentation on patients' progress was obtained. Information on the time of diagnosis and drug therapy was also obtained. Patient adherence to medications and individual treatment regimens were checked in patients' medical charts, and confirmed by their physicians. All relevant data were recorded in the questionnaire. Data collection was done over a 12-week period.

### 2.5 Outcomes

The primary outcome measure was association of patients' medication adherence with socio-demographic and clinical data, while the secondary outcome measures was glycaemic control.

### 2.6 Data collection tool

A three-part structured questionnaire was used to assess medication adherence of the study participants. Section A was used to obtain socio-demographic information of the respondents, while Section B obtained respondents' clinical characteristics and medications used (hospital records), and section C measured respondents' level of medication adherence. Obtained socio-demographic information included: patients age, gender, marital status, highest educational qualification, occupation and monthly income.

Section C had 7 medication adherence questions, and each question had a response scale of “yes” or “no”. Each “no” response was rated as “1” and each “yes” was rated as “0. Total scores ranged between 0 and 7 and this was categorised into three levels of adherence: high adherence (score = 7), medium adherence (score of 4 to <7), and low adherence (score < 4).

### 2.7 Validation and pretesting of data collection tool

The questionnaire content was assessed for validity through pretesting and assessment by 3 clinical pharmacists and 2 endocrinologists (face validity and content validity). The pretesting was conducted among 15 selected respondents who were not included in the study. The results from the process led to the modifications of some questions, to reduce ambiguity and ensure adequate comprehension by study participants.

### 2.8 Data analysis

Data entry and analysis were performed using SPSS version 24.0. Descriptive statistics were used for the demographic and clinical characteristics of the patients, including their medication adherence scores. The categorical variables were described by percentages and frequencies. Chi-square was used to evaluate associations between categorised variables with *P < 0.05* considered statistically significant. Cronbach's alpha was used to assess reliability of the questionnaire.

### 2.9 Ethical considerations

The study was conducted in line with institutional and review board ethics committee requirements. Ethical approval was obtained from the state hospital (Ijebu-Ode general hospital) ethics board with reference number: IT/1/VOLI. Informed consent was also obtained from the patients, and confidentiality was ensured.

## 3 Results

The questionnaire had a reliability of 0.71, while a total of 200 patients participated in the study, showing a response rate of 100%. The study participants comprised more of older adults of 60 years old and above 141 (70.5%) and majority were females 136 (68.0%), married 120 (60.0%) and not employed 69 (34.5%). More than half of the population had less than secondary education 102 (54.0%) and only few 37 (18.5%) earned above 50, 000 Naira (83.33 US dollars). Adherence to antidiabetic medications was not associated with any of the socio-demographic characteristics of the patients (*P > 0.05*). However, high adherence to medications was observed in only few of the patients, ≤ 2 (1%). See Table 1.

**Table 1:** Baseline Characteristics of diabetic patients according to Medication adherence scores *N* = 200.

Characteristics	Total	Low adherence	Moderate adherence	High adherence	*P*-value
Gender					
Male	64(32.0%)	24(12.0%)	40(20.0%)	0 (0.0%)	*P* = 0.55, X^2^ = 1.179
Female	136(68.0%)	55(27.5%)	79 (39.5%)	2 (1.0%)	

Age group					
<40	5 (2.5%)	2(1.0%)	3(1.5%)	0 (0.0%)	*P* = 0.522, X^2^ = 7.134, df = 8
40–49	14(7.0%)	2(1.0%)	9(4.5%)	1(0.5%)	
50–59	40(20.0%)	17(8.5%)	23(11.5%)	0 (0.0%)	
≥60	141(70.5%)	56(28.0%)	84(42.0%)	1 (0.5%)	

Marital status					
Single	2(1.0%)	0 (0.0%)	2(1.0%)	0 (0.0%)	*P* = 0.903, X^2^ = 2.172
Married	120(60.0%)	49(24.5%)	70(35.0%)	1(0.5%)	
Separated	8(4.0%)	4 (2.0%)	4 (2.0%)	0 (0.0%)	
Widowed	70(35.0%)	26(13.-%)	43(21.5%)	1(0.5%)	

Religion					
Christian	117(58.5%)	45(22.5%)	71(35.5%)	1 (0.5%)	*P* = 0.902, X^2^ = 203, df = 2
Muslim	83(41.5%)	34(17.0%)	48(24.0%)	1 (0.5%)	

Occupation					
Self-employed	63(31.5%)	31(15.5%)	32(16%)	0 (0.0%)	*P* = 0.335, X^2^ = 9.090
Employed	13(6.5%)	6(3.0%)	7(3.5%)	0 (0.0%)	
Retiree	41(20.5%)	12(6.0%)	29(14.5%)	0 (0.0%)	
Vocational	14(7%)	4(2.0%)	10 (5%)	0 (0.0%)	
Non-employed	69(34.5%)	26(13.0%)	41(20.5%)	2 (1.0%)	

Income Naira (US dollars)					
No steady income	133(66.5%)	52(26%)	79(39.5)	2(1.0%)	
<50, 000 (<131.06)	30(15%)	12(6.0%)	18(9.0%)	0 (0.0%)	*P* = 0.945, X^2^ = 1.696
50–125, 000 (131.06–327.65)	25(12.5%)	9(4.5%)	16(8.0%)	0 (0.0%)	
>125, 000 (>327.65)	12(6.0%)	6(3.0%)	6(3.0%)	0 (0.0%)	

Education					
None	51(25.5%)	21(10.5%)	29(14.5%)	1(0.5%)	*P* = 0.525, X^2^ = 5.147
Primary	57(28.5%)	24(12.0%)	32(16.0%)	1(0.5%)	
Secondary	49(24.5%)	22(11.0%)	27(13.5%)	0 (0.0%)	
Tertiary	43(21.5%)	12(6.0%)	31(15.5%)	0 (0.0%)	

^⁎^ One U.S. dollar is equivalent to 600.00 (Naira).

In Table 2, oral medications were shown to be mostly used alone 187(93.5%), than in combination with insulin 13(6.5%), but this did not significantly influence adherence (*P = 0.075*) and (*P > 0.05*) respectively. Metformin was most commonly used 199 (99.5%), followed by pioglitazone 100 (50.0%) and glimepiride 85 (42.5%), respectively. The use of dipeptidyl peptidase-4 inhibitors was not reported or found in the patients' hospital records. Majority of the patients did not have questions concerning their medicines 182(91%) and had moderate medication adherence 109(54.5%).

**Table 2:** Medicines prescribed for type 2 diabetes patients and adherence level N = 200.

Medicines	Total	Low adherence	Moderate adherence	High adherence	P –value
Treatment of diabetes					
Oral antidiabetic agents	187(93.5%)	70(35.0%)	115(57.5%)	2(1.0%)	P = 0.075, X^2^ = 5.179
Oral antidiabetic agents + Insulin	13(6.5%)	9(4.5%)	4(2.0%)	0 (0.0%)	

Medicines used					
Metformin	199(99.5%)	79(39.5%)	118(59.0%)	2(1.0%)	*P* = 0.710, X^2^ = 0.594
Glibenclamide	37(18.5%)	12(6. 0%)	25(12.5%)	0 (0.0%)	*P* = 0.467, X^2^ = 1.525
Glimepiride	85(42.5%)	34(17.0%)	50(25.0%)	1(0.5%)	*P* = 0.967, X^2^ = 0.067
Gliclazide	25(12.5%)	11(5.5%)	14(7.0%)	0 (0.0%)	*P* = 0.782, X^2^ = 0.491
Pioglitazone	100(50.0%)	39(19.5%)	61(30.5%)	0 (0.0%)	*P* = 0.352, X^2^ = 2.088

Questions on your medications?					
Yes	18(9.0%)	8(4.0%)	10(5.0%)	0 (0.0%)	*P* = 0.830, X^2^ = 0.372
No	182(91.0%)	71(35.5%)	109(54.5%)	2(1.0%)	

Patients' clinical characteristics showed that majority 159 (79.5%) had poor glycaemic control (>126 mg/dl), and duration of illness was not statistically associated with adherence (*P = 0.511*). Majority did not practice self-blood glucose monitoring 152(76.0%), but this was not associated with medication adherence (*p = 0.673*). Self-blood glucose monitoring (SBGM) and time of monitoring in relation to before or after meal, did not significantly influence adherence to medicines (*P* *=* *0.673* and *P* *=* *0.951* respectively) and majority monitored their blood glucose before meal 193(96.5%) and had moderate medication adherence 115(57.5%). Results also showed that blood glucose values obtained from self-monitoring was significantly associated with adherence to medicines (*P* *=* *0.001*). See Table 3.

**Table 3:** Clinical characteristics according to medication adherence scores.

Characteristics	Total	Low adherence	Moderate adherence	High adherence	P-value
Duration of illness in years					
<1	37(18.5%)	10(5.0%)	26(13.0%)	1(0.5%)	P = 0.511, X^2^ = 9.227
1–5.99	93(46.5%)	40(20.0%)	53(26.5%)	0 (0.0%)	
6–10.99	45(22.5%)	19(9.5%)	25(12.5%)	1(0.5%)	
11–15.99	15(7.5%)	8(4.0%)	7(3.5%)	0 (0.0%)	
16–20.99	3(1.5%)	0 (0.0%)	3(1.5%)	0 (0.0%)	
>21	7(3.5%)	2(1.0%)	5(2.5%)	0 (0.0%)	

Self-monitoring of blood glucose (SMBG)					
Yes	48(24.0%)	18(9.0%)	30(15.0%)	0 (0.0%)	P = 0.673, X^2^ = 0.791
No	152(76.0%)	61(30.5%)	89(44.5%)	2(1.0%)	

#Values of blood glucose tests (mg/dl)					
<100 (Normal)	41(20.5%)	10(5.0%)	29(14.5%)	2 (1.0%)	P = 0.001*, X^2^ = 17.796
100–125 (Pre-diabetes)	63(31.5%)	20(10.0%)	43(21.5%)	0 (0.0%)	
≥125 (Diabetes)	96(48.0%)	49(24.5%)	47(23.5%)	0 (0.0%)	

Value obtained (mg/dl)					
Before meal	193(96.5%)	76(38%)	115(57.5%)	2(1%)	P = 0.951, X^2^ = 100
After meal	7(3.5%)	3(1.5%)	4(2%)	0 (0%)	

# Classification according to American Diabetes Association.

* Statistically significant.

## 4 Discussion

Study participants comprised mostly of older adults of low socio-economic status and low educational qualifications. The study also found that medication adherence was not associated with any of the socio-demographic characteristics of the patients. Meanwhile a previous study suggested a link between patient demographic factors and medication adherence in type 2 diabetes.^19^ Low socio-economic condition has also been associated with prevalence of diabetes and pre-diabetes in another study.^3^ This may be attributed to several factors, ranging from low awareness of the disease to lack of resources for effective management. Meanwhile, a previous study positively associated older age with adherence to diabetes medications.^5^ Possible longer duration of the diseases in older people may likely encourage awareness and enhance adherence. Younger age was also reported to be positively associated with high adherence to diabetes medication in a previous study. ^20^

Oral medications were mostly prescribed for the patients and this is consistent with a previous study in Nigeria,^15^ and WHO guideline which reserves insulin therapy for third line use.^1^ The use of oral medications has also been seen to promote adherence in persons with type 2 diabetes mellitus as reported in a previous study.^5^ This shows that persons on insulin therapy are more likely to be non-adherent to their medicine.^10^ The major route of administration of insulin may also be a major limitation to adherence in patients. The most frequently prescribed medicines in this study were metformin and pioglitazone, but adherence was not significantly associated with type of medicines used.

Moderate adherence to medications was mostly observed among the respondents, while high adherence was rare. Several previous studies reported low medication adherence among Nigerian populations^16^^,^^17^^,^^21^ and Ethiopia.^22^ This was however, not consistent with findings from another study in Brazil,^20^ and Eastern Nigeria where medication adherence was reportedly high among study participants.^23^ Medication adherence is associated with glycaemic control,^23^ therefore high adherence is necessary for meeting glycaemic target and good diabetes outcomes.^20^ It is hence an essential component of diabetes care. The observed adherence level among the patients emphasises the need for enhanced medication information and counselling practices during routine clinic visits. Being an essential role of pharmacists and pharmacy technicians,^24^, ^25^, ^26^ medication information and counselling should be critical components of diabetic patients care.

Findings from the study showed that glycaemic control was not achieved in the majority of the patients. The observed moderate adherence in the study, may have resulted in this finding. Similarly, previous studies in South-west Nigeria^15^ and Ethiopia,^22^ also reported poor glycaemic control among persons with type 2 diabetes. However, this finding differed from a previous study in Eastern Nigeria where a high percentage of the respondents had good glycaemic control.^23^ This difference is likely associated with the difference in patient characteristics. Sub-optimal glycaemic control usually results from poor adherence to diabetic medications, and associated with poor clinical, economic and humanistic outcomes. This is often seen as poor disease prognosis with the development of macro and micro-vascular complications, reduced quality of life, increased cost of care, increased morbidity and mortality.^27^ Adherence to medications is associated with health-related parametres,^28^ therefore, it is of critical importance to achieving optimal disease management.

Clinical characteristics of the patients showed that duration of illness was not significantly associated with medication adherence. Meanwhile, duration of diabetes illness was associated with medication adherence in a previous study in Brazil.^20^ Difference in socio-demographic characteristics of the study participants may have influenced this difference in findings. Patient education and counselling are previously identified means of enhancing medication adherence. Also higher educational level, being mostly associated with improved awareness is another identified factor in improved medication adherence in chronic diseases,^15^^,^^16^ this however was found to be low in this group of patients. This is suggestive of low diabetes awareness in the patients, therefore urgent educational intervention on diabetes control is very relevant for this group.

Most of the patients did not monitor their blood glucose level. This was consistent with previous studies in Nigeria that reported poor practice of self-management^15^ and poor practice of blood glucose monitoring,^29^ but contrary to a study in Brazil were over half of the study participants practiced self-glucose monitoring.^20^ Patients are required to monitor and keep records of their own blood glucose measurements for enhanced outcomes.^30^ Meanwhile, diabetes awareness through routine education on self-care is an effective means of achieving patients' involvement in the disease management. Again the financial limitations that is likely associated with this population as seen in their baseline characteristics, may be a major limitation to self-glucose monitoring. Therefore, provisions could be made for the involvement of donor agencies in the give-away of self-glucose test kits to this category of persons at diabetic clinics.

Several influencing factors of adherence were seen in the study, one of which was results from self-blood glucose test. Findings showed that results of blood sugar self-test significantly influenced adherence to medicines among the study participants. Although glycaemic control is associated with adherence to treatment, its sustenance is vital for preventing diabetes-related complications.^27^ Meanwhile, fasting blood glucose tests was prevalent among the study participants who practiced self-monitoring, and the patients in this group predominantly had moderate medication adherence.

Some limitations were noted to be associated with this study following its self-reported component. Therefore, self-reporting bias is not to be over-looked in the interpretation of the study. However, this was minimised by the review of case notes for documented evidence. Being a single centre study also limits generalisability of results. Meanwhile, the study raises awareness on the medication adherence practices of persons with diabetes among the low socio-economic population, showing a clearer picture of the situation and its associated factors, for improved diabetes control plan. It provides additional evidence on the roles of medication adherence and its associated factors in the growing burden of diabetes in Nigeria.

## 5 Conclusions

Poor glycaemic control was prevalent among study participants, and oral antidiabetics were mostly prescribed. Moderate medication adherence was also mostly observed, and was associated with self-glucose monitoring. These findings suggest the necessity for targeted diabetes education on medication adherence, particularly during waiting time at the clinics, for enhanced medication adherence and improved diabetes control.

## Declaration of Competing Interest

The authors declare that they have no known competing financial interests or personal relationships that could have appeared to influence the work reported in this paper.

## Appendix A Supplementary data

Supplementary material Image 1
