# Supplementary material for: Drug therapy and medication adherence in type 2 diabetes in a care facility: A cross sectional survey
Source: Explor Res Clin Soc Pharm. 2022 Nov 4;8:100200. doi: 10.1016/j.rcsop.2022.100200 (PMC9667283; doi:10.1016/j.rcsop.2022.100200)
Supplement: Supplementary file 1 — Supplementary material [file mmc1.pdf]

DEPARTMENT OF CLINICAL PHARMACY AND BIO-PHARMACY

FACULTY OF PHARMACY OLABISI ONABANJO UNIVERSITY, SAGAMU CAMPUS OGUN STATE

Questionnaire on Drug therapy and medication adherence in type 2 diabetes in a care facility: A mixed methods study

Dear Respondents,

The purpose of this questionnaire is to assess drug therapy for type 2 diabetes, glycaemic control and association of medication adherence with socio-demographic and clinical data, among adult diabetic patients. Frank answers to all the questions will be appreciated, as all information will be treated as confidential. Participation is voluntary and does not include any monetary gain. Kindly append your signature below, if you agree to participate in this study.

Thank you for your co-operation.

I hereby agree to fill out this questionnaire.

____

Signature

Please indicate your opinion by ticking the appropriate information

SECTION A

Demographic Data

1. Age group:

[ ] 20-29yrs [ ] 30-39yrs [ ] 40-49yrs [ ] 50-59yrs [ ] 60-69yrs [ ] 70- 79yrs [ ] 80yrs &above

2. Gender: [ ]Male [ ]Female
3. Marital status: [ ] Single [ ] Married [ ] Separated [ ] Divorced [ ]Widowed
4. Religion: [ ] Christian [ ] Muslim [ ] Traditional
5. Highest educational qualification:

   [ ] None [ ] Primary school [ ] Secondary school [ ] Tertiary institution
6. Occupation: [ ] Student [ ] Self employed [ ] Employee/Civil servant

   [ ] Retiree [ ] Vocational [ ] Non-employed
7. What is your average monthly income? (Include pension, public assistance, support from family/friends, etc.)

   [ ] Less than ₦ 5,000 [ ] ₦ 5, 000 - 20,000 [ ] ₦ 20,000 - 35,000 [ ] N35,000 - 50,000

   [ ] More than ₦ 50, 000 [ ] cannot be quantified

Section B: Medications and clinical characteristics

1. When were you diagnosed with diabetes? Month ______ Year _____
2. Treatment for diabetes: [ ] Pills [ ] Insulin [ ] Pills and Insulin
3. What type of medications are you currently taking? [ ] Metformin

   [ ] Glibenclamide [ ] Glimepiride [ ] Gliclazide [ ] Pioglitazone

   [ ] Vidagliptin [ ] Sitagliptin
4. Do you have any question about your medication? [ ] Yes [ ] No

5. Do you test your blood sugar at home? [ ] Yes [ ] No
6. What value was last obtained? _______mg/dL
7. When was the last value obtained? [ ] Before meals [ ] After meals

Section C: Medication adherence

	**Questions**	**Yes**	**No**
1	Do you forget to take your diabetes medicines sometimes?		
2	Did you forget to take your diabetes medicine yesterday?		
3	Did you miss taking your medicine any day in the last 7 days?		
4	Do you stop taking your medicines because of suspected side effects?		
5	Do you stop taking your medicines for other reasons?		
6	Do you go days without taking your diabetes medicines?		
7	Do you alter the dosing of you diabetes medicines?		

Thank you.
